# High-frequency ventilation for acute traumatic and nontraumatic lung injury

**DOI:** 10.1186/cc12058

**Published:** 2013-03-19

**Authors:** R Varutti, R Bigai, M Fiorillo, D Tomasello, W Mercante, G Trillò

**Affiliations:** 1 Azienda Ospedaliera Santa Maria degli Angeli, Pordenone, Italy; 2 Udine University Hospital, Udine, Italy

## Introduction

ARDS is commonly observed in trauma patients. In some instances the severity of the clinical presentation is such that all conventional ventilatory support mode fails. In this setting, high-frequency oscillatory ventilation (HFOV) was considered mostly a rescue therapy.

## Methods

Fifteen adult patients admitted to our ICU for acute traumatic and nontraumatic lung injury were submitted to HFOV when conventional mechanical ventilation failed.

## Results

**Figure 1:**
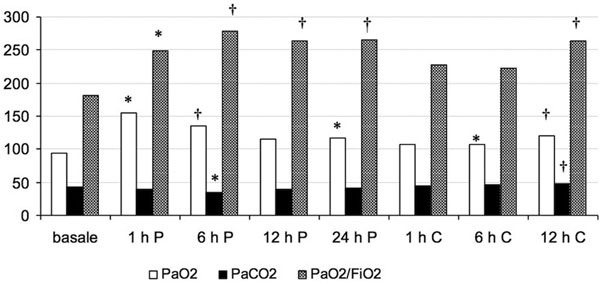
**Mean PaO_2_, PaCO_2_, PaO_2_/FiO_2 _at baseline and during HFOV (P, percussionator; C, conventional)**. *P <0.05 versus baseline, ^†^*P *<0.01 versus baseline.

Clinical and demographic data are shown in Table 1. Figure 1 shows the trend of gas parameters during the recovery. At baseline PaO_2 _was 94 ± 28 mmHg; after 6 hours of HFOV: 135 ± 41 mmHg, *P <*0.01. At baseline PaO_2_/FiO_2 _was 182 ± 97 mmHg; after 6 hours of HFOV: 264 ± 101 mmHg, *P *< 0.01. The benefits are maintained when returned to conventional ventilation.

**Table 1:** Clinical and demographic data

	Mean
Age (years)	64
Male/female	11/4
Dead	4
Trauma	3
SAPS II	45/40
APACHE	23/8

## Conclusion

HFOV may therefore be anticipated to improve end-organ perfusion and gas exchange; it should be considered in severe traumatic and nontraumatic respiratory failure [1].
